# Ramucirumab combination with sorafenib enhances the inhibitory effect of sorafenib on HepG2 cancer cells

**DOI:** 10.1038/s41598-022-21582-w

**Published:** 2022-10-25

**Authors:** Amna Mohamed Taha, Mohammad Mabrouk Aboulwafa, Hamdallah Zedan, Omneya Mohamed Helmy

**Affiliations:** 1 Egyptian Drug Authority, Wezaret Alzera’h St., Doki, Cairo, Egypt; 2 Department of Microbiology and Immunology, Faculty of Pharmacy, Ain Shams University, Al Khalifa Al Ma’moun St., Abbassia, Cairo, Egypt; 3 Department of Microbiology and Immunology, Faculty of Pharmacy, Cairo University, Kasr El-eini St., Cairo, Egypt; 4 Present Address: Faculty of Pharmacy, King Salman International University, Ras-Sudr, South Sinai Egypt

**Keywords:** Biochemistry, Cancer, Cell biology, Chemical biology, Molecular biology, Medical research, Molecular medicine, Oncology

## Abstract

Sorafenib, an oral multiple kinase inhibitor, is the standardized treatment for hepatocellular carcinoma (HCC). One strategy to improve HCC therapy is to combine agents that target key signaling pathways. In this study we set out to investigate the effect of combining sorafenib with either bevacizumab (anti-VEGF), panitumumab (anti-EGFR) or ramucirumab (anti-VEGFR2) on HepG2 cancer cell line with the aim of improving efficacy and possibility of therapeutic dose reduction of sorafenib.: HepG2 cancer cell line was treated with sorafenib alone or in combination with either bevacizumab, panitumumab or ramucirumab. Cell proliferation; apoptosis and cell cycle distribution; gene expression of *VEGFR2, EGFR, MMP-9* and *CASPASE3*; the protein levels of pVEGFR2 and pSTAT3 and the protein expression of CASPASE3, EGFR and VEGFR2 were determined. Combined treatments of sorafenib with ramucirumab or panitumumab resulted in a significant decrease in sorafenib IC_50_. Sorafenib combination with ramucirumab or bevacizumab resulted in a significant arrest in pre-G and G0/G1 cell cycle phases, significantly induced apoptosis and increased the relative expression of *CASPASE3* and decreased the anti-proliferative and angiogenesis markers´ *MMP-9* and pVEGFR2 or *VEGFR2* in HepG2 cells. A significant decrease in the levels of pSTAT3 was only detected in case of sorafenib-ramucirumab combination. The combined treatment of sorafenib with panitumumab induced a significant arrest in pre-G and G2/M cell cycle phases and significantly decreased the relative expression of EGFR and *MMP-9*. Sorafenib-ramucirumab combination showed enhanced apoptosis, inhibited proliferation and angiogenesis in HepG2 cancer cells. Our findings suggest that ramucirumab can be a useful as an adjunct therapy for improvement of sorafenib efficacy in suppression of HCC.

## Introduction

Hepatocellular carcinoma (HCC) is the fifth most common cancer worldwide, accounting for 75–85% of liver cancer’s cases; it is often diagnosed in late stages when most therapeutic options are not very effective^1^. It is the second leading cause of cancer deaths worldwide and is associated with substantial morbidity and mortality; World Health Organization estimates that more than one million patients will die from liver cancer in 2030^2^. Among the important factors for HCC development are angiogenesis and signaling cascades for regulating cell proliferation such as the Raf/MAPK kinase/MAPK pathway, which is activated in numerous hepatic carcinoma derived cell lines and tumor samples^3^. Tumor angiogenesis is induced by an increased secretion of endogenous angiogenic factors or down-regulation of angiogenesis inhibitors^4,5^. Angiogenic factors are produced by tumor cells and by other cell types such as: endothelial cells, fibroblasts, smooth muscle cells/pericytes, and infiltrating immune cells; they initiate the angiogenic process by activating endothelial cells and induction of the angiogenic switch^4,6^. Vascular endothelial growth factor (VEGF) is involved in endothelial cell activation and is the major tumor angiogenesis factor^6,7^. Inhibiting endothelial cell growth and survival through the use of endothelial-specific integrin inhibitors, or inhibiting endothelial cell invasion using specific inhibitors of MMPs are used approaches to hinder tumor growth and spread^8^.

Sorafenib, an oral multiple kinase inhibitor, is the first molecular targeted therapy approved by the U.S. Food and Drug Administration (FDA) for advanced HCC, and is now its standardized treatment^3,9^. It significantly inhibits the activities of multiple tyrosine and serine/threonine kinases, as well as tumor angiogenesis, cell proliferation and induces apoptosis^9^. Sorafenib inhibits tumor angiogenesis by targeting Raf serine/threonine kinases, besides different receptor tyrosine kinases, including c-Kit, FLT-3, vascular endothelial growth factor (VEGF) and platelet-derived growth factor (PDGF) signaling^10^. However, in clinical practice, sorafenib produces relatively low tumor response rates in the majority of HCC patients and is beneficial in only ~ 30% of the patients^3^. Sorafenib treatment has severe toxicity and many adverse reactions; this pushes a large percentage of patients to reduce sorafenib dose or terminate treatment^3^. Even with treatment, the survival of certain patients is short^3^; also in most patients, who initially responded to sorafenib, tumor recurrence and progression often occurs following a few months of sorafenib therapy^3^.

Different compounds were tested for their suitability, as a combination therapy with sorafenib, to overcome its lower efficacy treatment of advanced HCC, including bortezomib, rapamycin and a selective MEK inhibitor, AZD6244; the combined therapies had better responses compared to sorafenib^11^. Combining therapies that inhibit different signaling pathways has the potential to be more effective than a single pathway inhibition and to overcome tumor resistance^12^. Vascular endothelial growth factor (VEGF) and epidermal growth factor receptor (EGFR) inhibitors are fundamental treatments for several tumor types^13,14^. Bevacizumab is a recombinant humanized monoclonal antibody that binds VEGF with high affinity^15^, thereby inhibiting tumor growth, paracrine/autocrine growth factor release and metastasis^15^. Bevacizumab, as a single agent or in combination with other agents, has shown modest activity in treating advanced HCC^16^. Ramucirumab is a fully humanized monoclonal antibody that targets the extracellular domain of VEGFR-2 and is administered intravenously every 2 or 3 weeks^17,18^. EGFR is a tyrosine kinase transmembrane receptor that is overexpressed in several types of cancer cells; it plays a central role in tumor proliferation^12^, survival and differentiation^13,19^. Panitumumab is a fully humanized antibody, IgG2, that binds to EGFR and prevents receptor dimerization, tyrosine autophosphorylation of EGFR and the activation of downstream signaling molecules^13^. In clinical trials, combining bevacizumab, an anti-VEGF monoclonal antibody (mAb), with either cetuximab, an anti-EGFR mAb, or erlotinib, an EGFR tyrosine kinase inhibitor, had an increased antitumor activity compared with treating with either of these anti-EGFR agents alone or in combined chemotherapy^20^. Therefore, inhibition of both pathways could improve the antitumor efficacy and overcome resistance to EGFR inhibition^21,22^.

Resistance to anti-VEGF therapy can be mediated via the overexpression of VEGF receptors, an increase in VEGF levels, and the upregulation of alternative angiogenesis signaling pathways, such as platelet-derived growth factor receptor (PDGFR) signaling^23,24^. Therefore, a complete blockage of the VEGF-signaling pathway by combining a ligand inhibitor, such as bevacizumab, with a multi-targeted kinase inhibitor, blocking the VEGF system on the receptor level (sorafenib) and also targeting compensatory pro-angiogenic mechanisms, could cause a synergistic inhibition of tumor angiogenesis. Thus, combining sorafenib with other antiangiogenic agents, with different targets, may improve the efficacy of sorafenib monotherapy and minimize the development of drug resistance. Combining sorafenib with different targeted therapies would allow dose reduction of sorafenib, without concomitant loss of its effectiveness, and thus reducing its toxicity and overcome tumor resistance. This could complement bevacizumab activity by the complete vertical blocking of VEGF signaling and inhibiting other angiogenic pathways potentially involved in mediating resistance to bevacizumab^23^.

A synergistic combination treatment plan that includes sorafenib at low doses, to decrease its toxicity, along with an anti-VEGF, anti-EGFR or an anti-VEGFR2 could result in a better cytotoxic effect in case of HCC. We set up to investigate the therapeutic efficacy of different combinations of sorafenib with bevacizumab, or panitumumab or ramucirumab, on the hepatic cancer cell lines HepG2. Several parameters were tested, including cellular proliferation, cell cycle regulation, apoptosis, relative expression of *VEGFR2, EGFR, MMP-9* and *CASPASE3* and the protein levels of pVEGFR2 and pSTAT3 in HepG2 cancer treated cells. Sorafenib-ramucirumab was proved to be the optimum combination that would allow dose reduction of sorafenib, without concomitant loss of its effectiveness, and thus lessening its toxicity.

## Materials and methods

### Tested therapeutic agents

Sorafenib powder was kindly provided by the National Organization for Drug Control and Research (NODCAR, Cairo, Egypt). The tested monoclonal antibodies included: bevacizumab (Avastin®; 25 mg/mL, Genetech, Switherland), panitumumab (Vectibix®; 20 mg/mL, Amgen, USA) and ramucirumab (Cyramza®; 10 mg/mL, Lilly, USA). Sorafenib was dissolved in dimethyl sulfoxide (DMSO; Sigma- Aldrich, USA) to a final concentration of 10 mM; the monoclonal antibodies were diluted in RPMI 1640 medium to the tested concentrations chosen from literature^25–28^.

### Culturing and treating HepG2 with the tested therapeutic agents

Hepatocellular carcinoma cell line (HepG2), (ATCC® HB-8065; Manassas, VA, USA), were cultured and maintained in Roswell Park Memorial Institute (RPMI) 1640 medium (Gibco; Thermo Fisher Scientific, USA) supplemented with 10% fetal bovine serum (FBS; Gibco; Thermo Fisher Scientific, USA), 10,000 U/mL penicillin/10 mg/mL streptomycin and incubated at 37 °C in a humidified incubator with 5% CO_2_, according to the supplier.

### Determination of the IC50 of sorafenib, bevacizumab, panitumumab and ramucirumab and their combinations on HepG2 cancer cells using MTT assay

The effect of sorafenib, bevacizumab, panitumumab and ramucirumab as well as their combinations on HepG2 cell viability was determined using MTT assay^29^. HepG2 cancer cells cultured in 96-well plates (Costar^Ⓡ^, Corning, Switzerland) were treated with 100 µl of two fold serially diluted solutions of the tested agents in RPMI and the plates were incubated at 37 °C in a humidified incubator with 5% CO_2_. Sorafenib was tested at concentrations ranging from 50 to 0.1 µM for 24, 48 and 72 h treatment periods, while the monoclonal antibodies were tested at concentrations ranging from 2000 to 0.98 µg/mL for 48 and 72 h treatment periods. For testing combinations of sorafenib with the monoclonal antibodies, sorafenib was used at concentrations ranging from 0.1 to 50 µM while the monoclonal antibodies were tested at concentrations ranging from 500 to 62.5 µg/mL for 48 h; untreated HepG2 cells were used as a control.

The absorbance was determined at 490 nm using a microplate reader (Bio Tek, Winooski, VT, USA). The assay was repeated three times independently. The percentage of viability relative to control was calculated using the following formula: Viability (%) = (A_490_ of treated cells/A_490_ of control cells) × 100^29^. Dose response curves for assessing the effect of single or combination treatment were constructed. Half-maximal inhibitory concentration (IC50), the drug concentration at which 50% growth inhibition occurs, was calculated using GraphPad Prism software, version 6.00 (GraphPad Software, Inc. La Jolla, CA, USA). The combination index value was calculated from the fraction-affected value of each combination, according to the Chou–Talalay method, using CompuSyn software (ComboSyn, Inc.). A combination index value below 1 indicates synergism^30^.

### Microscopial examination of the morphological and structural changes in HepG2 cancer treated cells

HepG2 cancer cells were cultured in tissue culture flasks (TCF), 25 cm^2^ surface area cell culture. For testing individual agents, the cultured cells were treated with: IC_50_ of sorafenib, 250 µg/mL, each of bevacizumab, panitumumab or ramucirumab. For testing sorafenib combinations with each monoclonal antibody, the IC_50_ of sorafenib was used with 250 µg/mL of the tested monoclonal antibody. The flasks were incubated for 48 h at 37 °C in a humidified incubator with 5% CO_2_. Thereafter, the cells were harvested by trypsinization and fixed gently by cold alcohol and stained by Hematoxylin and Eosin for histopathological examination^31^.

### Flow cytometry for testing the effect of sorafenib, bevacizumab, panitumumab and ramucirumab and sorafenib-monoclonal antibody combinations on cell cycle and apoptosis in HepG2 cancer cells

HepG2 cancer cells were cultured in tissue culture flasks (TCF). The cultured cells were treated with one of the following: IC_50_ of sorafenib, 250 µg/mL bevacizumab, 250 µg/mL panitumumab, 250 µg/mL ramucirumab and the IC_50_ of sorafenib with 250 µg/mL of the tested monoclonal antibody; the flasks were incubated for 48 h at 37 °C in a humidified incubator with 5% CO_2_.

Cell cycle analysis, to determine the distribution of cells in the different cell cycle phases (G0/G1, S and G2/M), by measuring the DNA content of the nuclei labeled with propidium iodide^32^, was performed using flow cytometry^32^. In brief, cells were harvested by trypsinization and washed with cold phosphate-buffered saline (PBS); fixed with 70% ethanol at 4 °C for 24 h; then re-suspended in PBS containing 40 μg/mL propodium iodide (Sigma-Aldrich), 0.1 mg/mL RNase and 0.1% (v/v) Triton X-100 in a dark room for 30 min. The fluorescence intensity of individual cells was measured by a flow cytometer (Becton Dickinson, San Jose, CA).

For apoptosis analysis, the treated cells were double stained using FITC Annexin-V apoptosis detection kit (BioVision, Palo Alto, CA), according to manufacturer's protocol. Annexin V-FITC binding was detected by flow cytometry^33^. Each sample was assayed three times. The coefficient of drug interaction (CDI) was calculated as follows: CDI = AB/(A * B), where AB is the ratio of the combination group to the control group; A or B is the ratio of the single agent group to the control group. A CDI value ˂ 1, = 1 or ˃ 1 indicates synergistic, additive or antagonistic effects, respectively^34^.

### RT-PCR for determining the relative expression of VEGFR2, EGFR, MMP-9 and CASPASE3 genes in HepG2 cancer treated cells

Total RNA extraction from cultured HepG2 cancer cells, treated with IC_50_ of sorafenib, 250 µg/mL bevacizumab, 250 µg/mL panitumumab, 250 µg/mL ramucirumab and IC_50_ of sorafenib with 250 µg/mL of the tested monoclonal antibody, was performed using Gene JET RNA Purification kit (Thermofisher Scientific, EU, catalogue number K0731) according to the manufacturer’s instructions. The concentration and integrity of RNA were assessed using NanoDrop 2000 (Thermo Fisher Scientific, Waltham, MA, USA). cDNA was synthesized from 1 µg of total RNA using a Quantitect Reverse Transcription kit (Qiagen, Germany, catalogue number 205311), according to the manufacturer’s instructions.

Quantitative real-time PCR was performed using QuantiTect SYBR Green PCR kit (Qiagen, Germany, catalogue number 204343) using a Rotor-Gene Q cycler (Qiagen, Germany). RT PCR mixture consisted of 12.5 µl 2 × SYBR Green PCR Master Mix, 1 µl of each primer (10 pmol/µl), 2 µl cDNA and 8.5 µl RNase-free water in a final volume of 25 µl. The nucleotide sequences of the used primers are listed in Table 1. The amplification conditions included initial 10 min at 95 °C, followed by 40 two-step cycles of 15 s at 95 °C and 1 min at 60 °C and a final extension step at 60 °C for 10 min. Melting curves were performed after each run to determine the specificity of the tested primers. Relative fold changes in the expression of target genes (*VEGFR2, EGFR, MMP-9* and *CASPASE3)* were determined using the comparative 2^(−ΔΔCt)^ method with *GAPDH* as an internal control to normalize the level of target gene expression^35^. ΔΔ^CT^ is the difference between the mean Δ^CT^ (of treated HepG2 cancer cells) and mean Δ^CT^ (untreated HepG2 cancer cells), where Δ^CT^ is the difference between the mean CT for gene of interest and the mean CT for internal control gene in each sample.

**Table 1:** Sequences of RT-PCR primers used in the study.

Test gene	Primer	Sequence (5′–3′)
*CASPASE3*	*Cas3*	F -TTC ATT ATT CAG GCC TGC CGA GG
		R-TTC TGA CAG GCC ATG TCA TCC TCA
*VEGFR2*	*VEGFR*	F-CAGTGGGCTGATGACCAAGA
		R-GGGTGGGACATACACAACCA
*EGFR*	*EGFR*	F -TGACTCCGTCCAGTATTGATCG
		R -GCCCTTCGCACTTCTTACACTT
*MMP-9*	*MMP-9*	F-ATCCAGTTTGGTGTCGCGGAGC
		R-GAAGGGGAAGACGCACAGCT
*GAPDH*	*GAPDH*	F-GAAGGTGAAGGTCGGAGTCA
		R-TTGAGGTCAATGAAGGGGTC

### Determining the concentration of pSTAT3 and pVEGFR2 in HepG2 cancer treated cells

HepG2 cancer cultured cells, treated with IC_50_ of sorafenib, 250 µg/mL bevacizumab, 250 µg/mL panitumumab, 250 µg/mL ramucirumab and IC_50_ of sorafenib with 250 µg/mL of the tested monoclonal antibody were tested for their protein levels of pSTAT3 and pVEGFR2. For measuring the concentration of pVEGFR2, a sandwiched ELISA assay was used using phospho-VEGFR2 (Tyr1175) CISBIO kit (catalogue number 63ADK041PEG), according to the manufacturer’s instructions. The determination of pSTAT3 (pTyr705) concentration was carried out using RayBio® Phosphotyrosine STAT3 ELISA Kit (Ray Biotech, catalogue number PEL-Stat3-Y), according to the manufacturer’s instructions. Each assay was repeated three times.

### Protein extraction and western blot analyses for determining the protein expression of VEGFR2, EGFR and cleaved CASPASE3 in HepG2 cancer treated cells

Cultured HepG2 cancer cells, treated with the IC_50_ of sorafenib, 250 µg/mL bevacizumab, 250 µg/mL panitumumab, 250 µg/mL ramucirumab and the IC_50_ of sorafenib combined with 250 µg/mL of the tested monoclonal antibody, were lysed in modified RIPA buffer^36^. The protein concentration was determined using Bradford Protein assay Kit ((Bio Basic Inc., Markham, Canada), according to the manufacturer’s instructions. Twenty micrograms proteins were separated by sodium dodecyl sulfate polyacrylamide gel electrophoresis (SDS-PAGE) (BioRad, USA). Following electrophoresis, the separated proteins were transferred onto a PVDF membrane (Bio-Rad Laboratories, Inc., CA, USA). Membranes were blocked in tris-buffered saline with 0.1% Tween 20 (TBST) buffer and 3% bovine serum albumin (BSA), at room temperature for 1 h and then incubated with the following antibodies: anti- cleaved- CASPASE3 (ASP 175) (catalogue number PA5-114687) (Thermos Fisher Scientific, USA), anti-VEGFR2 (catalogue number BS-10412R) (Bioss, USA), anti-EGFR (catalogue number MA513070) (Thermos Fisher Scientific, USA) and anti-GAPDH (catalogue number AM4300), according to the manufacturer’s instructions. Subsequently, the membrane was probed with horseradish-peroxidase-conjugated secondary antibody (Thermo Fisher Scientific, USA) for 1 h at room temperature. The bands were visualized by a chemiluminescent substrate, Clarity™ Western ECL substrate (BIO-RAD, USA) and photographed with a ChemiDoc MP imager (BIO-RAD, USA). The band of the housekeeping control protein GAPDH was used to normalize the band intensity of the target proteins using the Chemi Doc MP imager software.

### Statistical analysis

All experiments were carried out in three replicates. Data were expressed as the mean ± standard deviation [SD] and analyzed using one-way analysis of variance [ANOVA] between more than one group and unpaired t test with Welch's correction for comparison between two groups. These statistical analyses were conducted by by GraphPad Prism 6 (GraphPad Prism software, La Jolla, CA, USA. The results were considered statistically significant at P < 0.05.

## Results

### Effect of sorafenib, bevacizumab, panitumumab and ramucirumab on viability of HepG2 cancer cell line using MTT assay

Treatment with sorafenib at concentrations up to 0.78 µM produced no significant effect on HepG2 cancer cells viability at either 24, 48 or 72 h treatment periods; sorafenib concentrations ranging from 1.56 to 50 µM produced a gradual decrease in cell viability which was both concentration and time dependent in most cases (S1 Fig). A 48 h treatment period with the tested monoclonal antibodies: bevacizumab, panitumumab and ramucirumab caused either no or little change in cell viability (S2A Fig); upon prolonging the treatment period to 72 h, a gradual decrease in cell viability was observed with both panitumumab and ramucirumab which was more pronounced at the higher tested concentrations. On the other hand, bevacizumab showed nearly no effect on HepG2 cell viability at its all the tested concentrations (S2B Fig).

**Figure 1:**
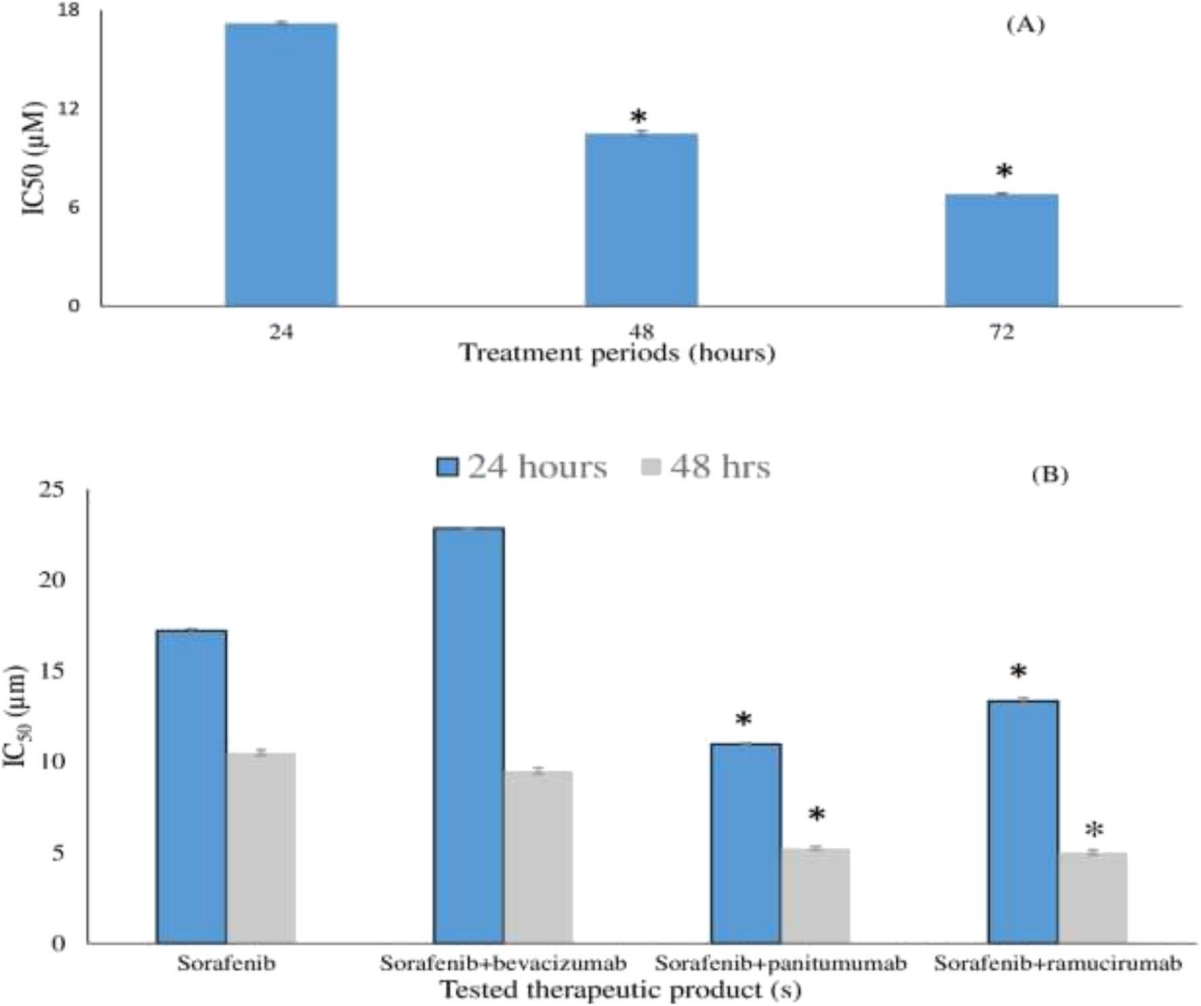
Effect of sorafenib and the combinations treatments on HepG2 cancer cells. HepG2 cancer cells were seeded in 96-well plates and treated with sorafenib in presence and absence of bevacizumab, panitumumab and ramucirumab. Each value represents the mean ± SD (n = 3). *P < 0.05 compared to sorafenib. (**A**) IC50 of sorafenib at the three treatment periods calculated using GraphPad Prism software. (**B**) Change in sorafenib IC_50_ in the presence of the tested monoclonal antibodies following 24 and 48 h treatment periods.

The IC_50_ of sorafenib decreased significantly upon prolonging the treatment period, P < 0.05 (Fig. 1A). By assessing the concentration–response curves of sorafenib cytotoxicity assays using the different tested combinations on HepG2 (S3 Fig), there was a significant change in the IC50 of sorafenib when combined with 250 or 500 µg/mL of either panitumumab or ramucirumab compared to single sorafenib treatment. The calculated combination indices for most of tested combinations showed synergism (CI ˂ 1) with sorafenib combinations with either 250 or 500 µg/mL of the tested monoclonal antibodies. (S1 Table). Hence the 250 µg/mL concentration of each tested monoclonal antibody was used in further experiments (Fig. 1B). Addition of panitumumab or ramucirumab to the tested concentration of sorafenib caused a significant time dependent decrease in sorafenib IC_50_, P < 0.05 (Fig. 1B). On the other hand, addition of bevacizumab to the tested concentrations of sorafenib caused nearly no effect following a 48-h treatment period and an increase in IC_50_ of sorafenib following a 24-h treatment period (Fig. 1B).

## Microscopial examination of the morphological and structural changes in HepG2 cancer treated cells

**Figure 2:**
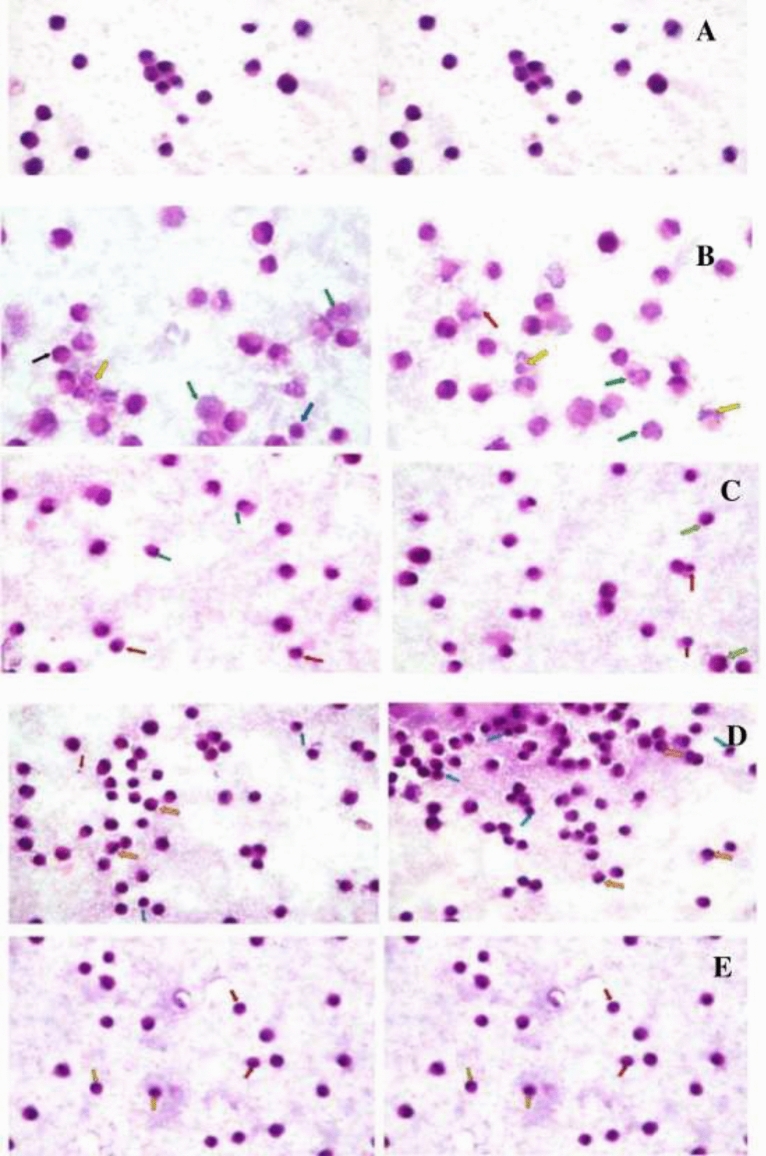
Photomicrographs showing the pathological changes occuring in HepG2 cancer cells after individuals treatments using 100 × magnification. The pathological changes were as follows: (**A**) untreated HepG2 cells (control) showing regular cells with hyperchromatic nuclei and nuclear pleomorphism; (**B**) Sorafenib treated cells showing nuclear fragmentation (yellow arrows), necrotic cells with mixed euochromatin and heterochromatin (green arrows), ruptured cell membrane (red arrow), peripheral condensation of chromatin (black arrow) and shrunken apoptotic cells (blue arrow); (**C**) Bevacizumab treated cells showing apoptotic features of shrunken cells, shrunken nuclei, shrunken apoptotic cells (red arrows) and peripheral condensation of chromatin (green arrows); (**D**) Panitumumab treated cells showing shrunken apoptotic (blue arrows) with peripheral condensation of chromatin (orange arrows) and apoptotic body (red arrow), and (**E**) Ramucirumab treated cells showing cellular and nuclear shrinkage (yellow arrow), irregularities of cellular and nuclear outlines (green arrows), peripheral condensation of chromatin (red arrow) and membrane blebbing (blue arrow).

**Figure 3:**
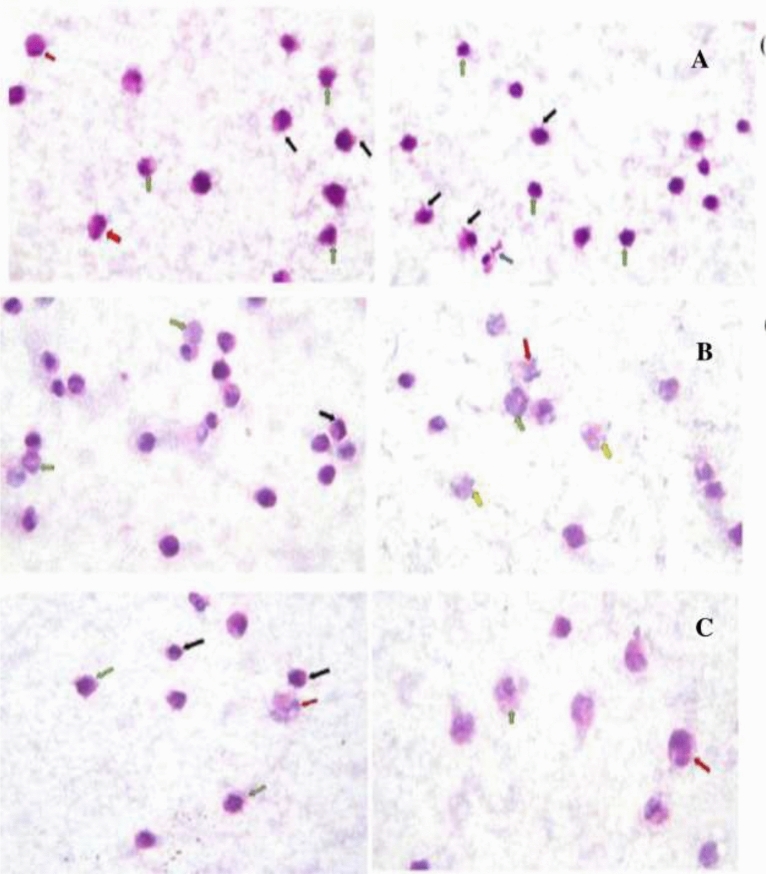
Photomicrographs showing the pathological changes occurring in HepG2 cancer cells treated with sorafenib’s combinations using 100 × magnification. The pathological changes were as follows: (**A**) Sorafenib-bevacizumab combination showing: swollen necrotic cells with mixed euchromatin and heterochromatin (Red arrows), irregularities of cellular and nuclear outlines (green arrows), ruptured cell membrane (Blue arrow) and membrane blebbing (Black arrows); (**B**) Sorafenib-panitumumab combination showing: ruptured cell membranes (yellow arrows), swollen necrotic cells with mixed euochromatin and heterochromatin (blue arrow), ruptured cellular membranes (Red arrow), necrotic cells with mixed euochromatin and heterochromatin (Green arrows) and peripheral condensation of chromatin (black arrow); (**C**) Sorafenib-ramucirumab combination showing swollen necrotic cells with mixed euchromatin and heterochromatin (blue arrow), ruptured cellular and nuclear membranes (red arrow), irregular cell membranes (green arrows) and shrunken apoptotic cells with irregular cellular and nuclear membranes (black arrows). *These pathological changes were in addition to those occured by each of the tested therapeutic product alone.

The observed morphological and structural changes in HepG2 cancer cells treated with sorafenib, bevacizumab, panitumumab and ramucirumab included: necrosis, nuclear fragmentation, ruptured membrane, apoptosis, shrunken nuclei and peripheral condensation of chromatin (Fig. 2). Combination of sorafenib with bevacizumab, panitumumab or ramucirumab showed extra pathological changes, including: swollen necrotic cells, ruptured cellular membranes besides irregularities in cellular and nuclear outlines and membrane blebbing (Fig. 3).

### Flow cytometry for testing the effect of sorafenib, bevacizumab, panitumumab and ramucirumab and sorafenib-tested monoclonal antibody combinations on cell cycle and apoptosis of HepG2 cancer cells

**Figure 4:**
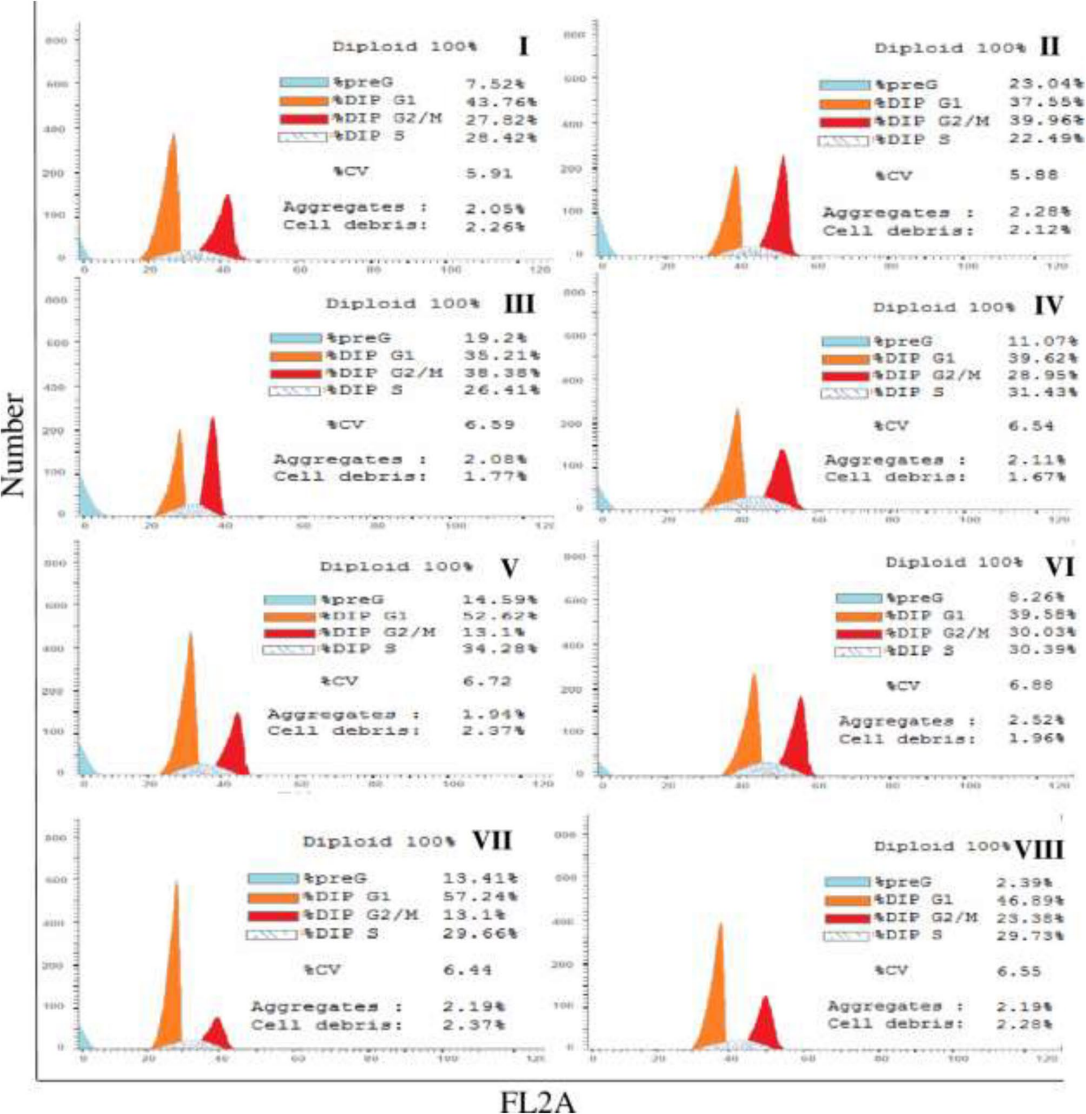
Flowcytometry cell cycle pattern analysis of HepG2 cancer cells. Treatments were as follows: sorafenib (I), bevacizumab (II), panitumumab (III), ramucirumab^12^, sorafenib-bevacizumab combination (V), sorafenib-panitumumab combination (VI), sorafenib-ramucirumab (VII) and untreated (control) HepG2 cancer cells (VIII).

Flow cytometry cell cycle analyses of the effect of the IC_50_ of sorafenib on HepG2 cancer cells in the presence and absence of 250 µg/mL of bevacizumab, panitumumab, or ramucirumab following 48 h treatment periods are shown in Fig. 4. Single treatment with sorafenib caused cell growth arrest in G2/M phase accompanied by an increase in the PreG1. This effect was also observed in case of single treatments by ramucirumab, bevacizumab and panitumumab. Sorafenib combination with bevacizumab caused a cell growth arrest in G0/G1, S and PreG1 phases; combination with panitumumab caused cell growth in G2/M and PreG1 and the combination with ramucirumab caused cell growth arrest in G0/G1 and PreG1 phases.

**Figure 5:**
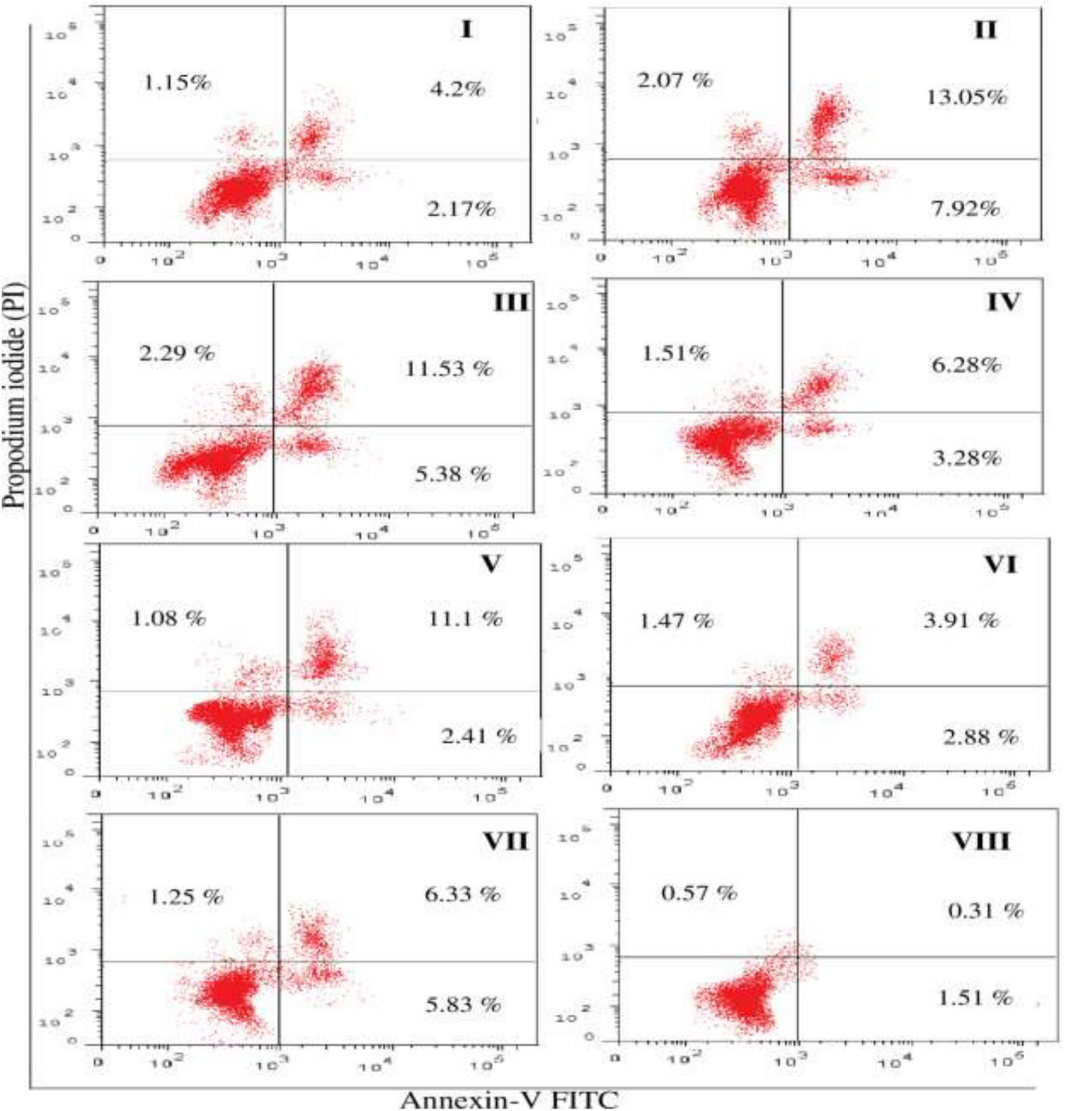
Flow cytometry analysis showing apoptosis and necrosis in HepG2 cancer cells. Treatments were as follows: sorafenib (I), bevacizumab (II), panitumumab (III), ramucirumab (IV), sorafenib-bevacizumab combination (V), sorafenib-panitumumab combination (VI), sorafenib-ramucirumab (VII) combination and untreated (control) HepG2 cancer cells (VIII).

The effect of treatments on the early signal transduction events, late morphological changes in cell size and DNA degradation together with necrosis occurring in HepG2 cancer cells are shown in Fig. 5. Treatment with single therapeutic agents resulted in total apoptosis in HepG2 cancer cells ranging from 7 to 23% with the highest effect recorded with bevacizumab and panitumumab. Combinations of sorafenib with either bevacizumab or ramucirumab recorded a higher apoptotic percentage than that recorded in case of sorafenib alone; nearly no change was recorded in case of sorafenib combination with panitumumab. Necrosis was not recorded by more than 3% in all treated HepG2 cancer cells by single or combined treatments.

### Effect of single and combined treatments on the relative expression of CASPASE3, MMP-9, VEGFR2 and EGFR in HepG2 cancer treated cells

**Figure 6:**
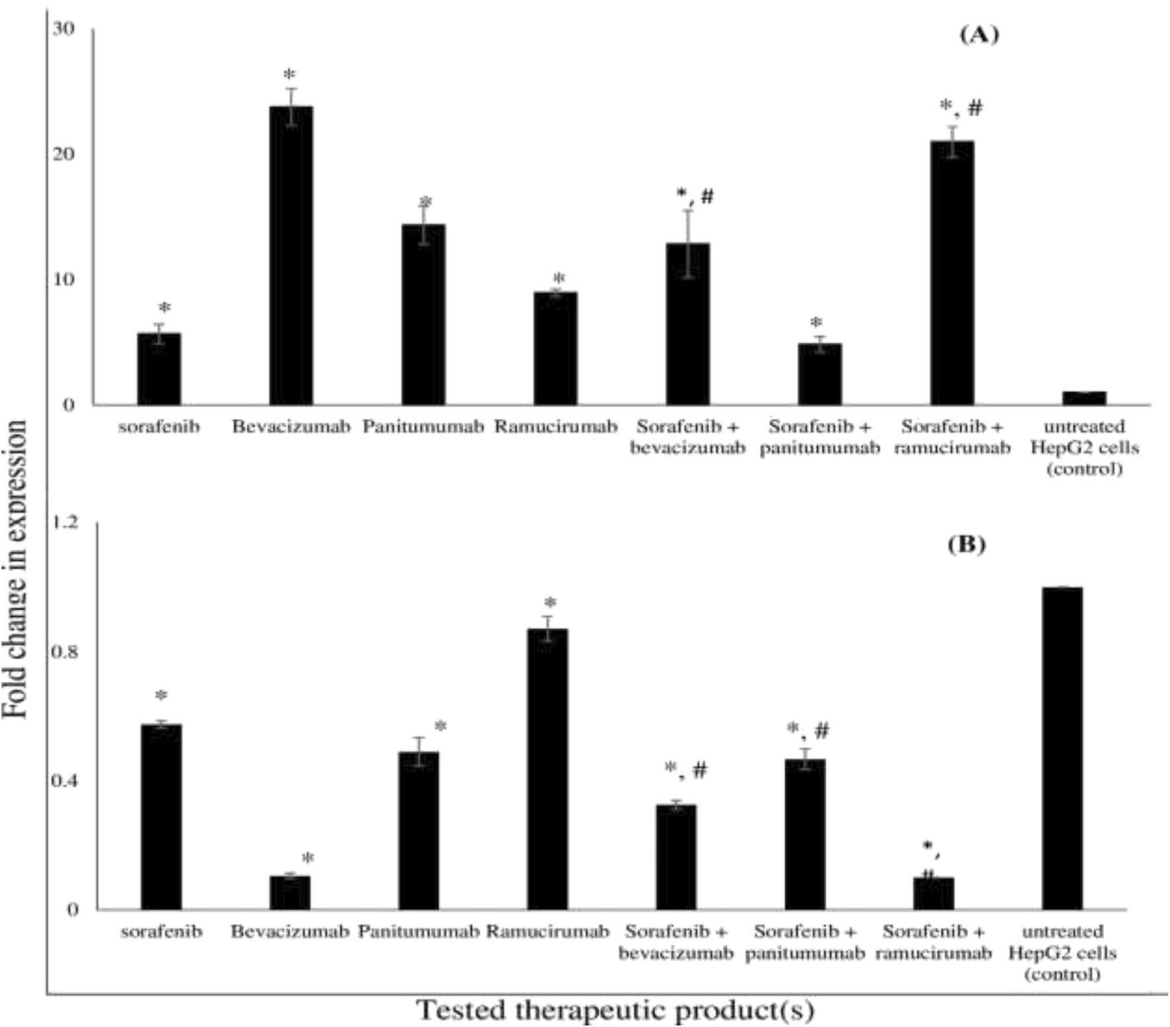
*CASPASE3* and *MMP-9* relative gene expression in HepG2 cancer treated cells. Effect of treatment with sorafenib in presence and absence of bevacizumab, panitumumab and ramucirumab on the expression of *CASPASE3* (**A**) and *MMP-9* (**B**) in HepG2 cancer cells determined by RT-PCR. Each value represents the mean ± SD (n = 3). *P < 0.05 compared to HepG2 untreated cancer cells (control). #P < 0.05, compared to sorafenib treated HepG2 cancer cells.

**Figure 7:**
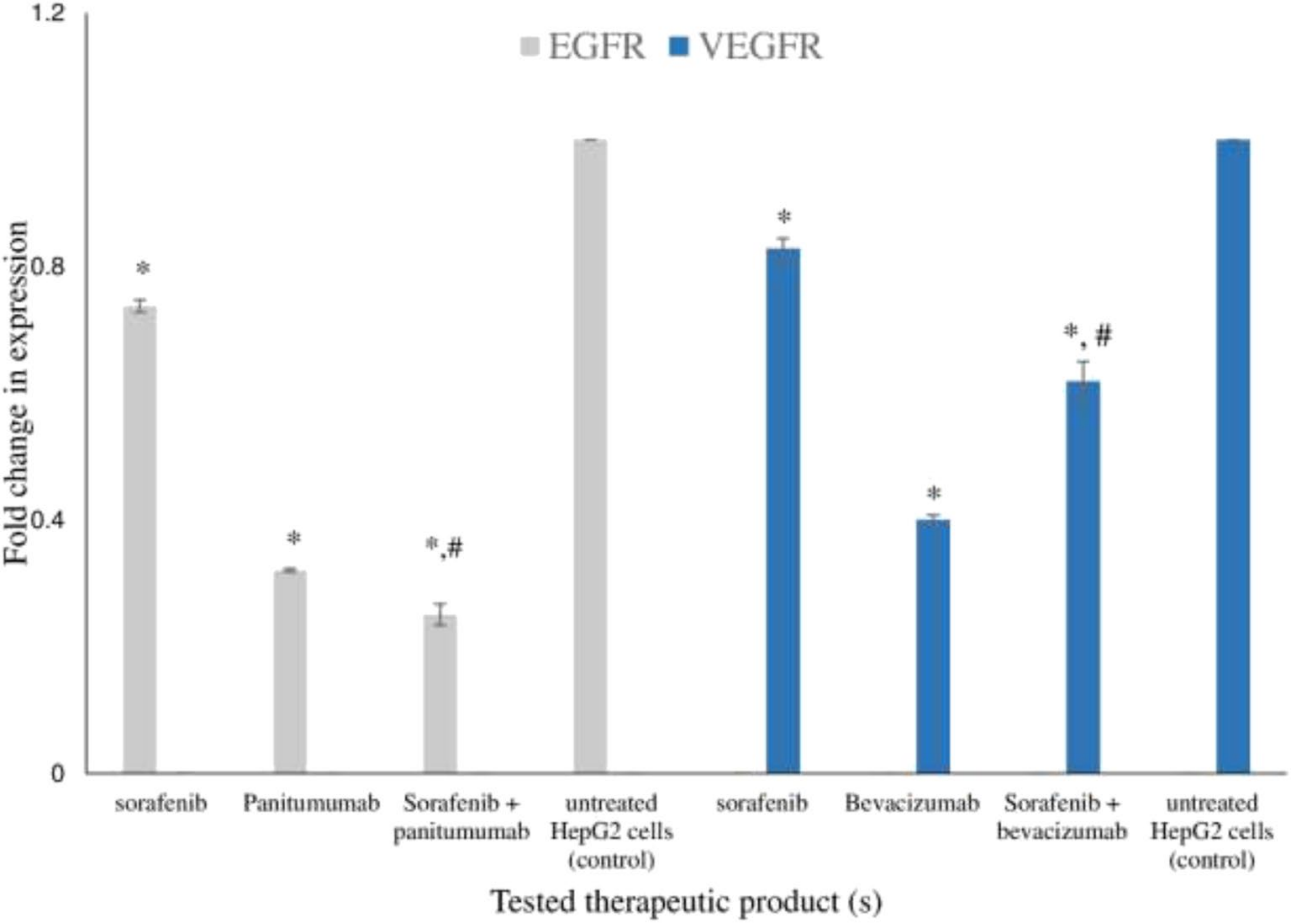
*VEGFR2* and *EGFR* relative gene expression in HepG2 cancer treated cells. The effect of treatment with sorafenib in the presence and absence of bevacizumab on the relative expression of *VEGFR2* and in the presence and absence of panitumumab on the relative expression of *EGFR* in HepG2 cancer cells determined by RT-PCR. Each value represents the mean ± SD (n = 3). *P < 0.05 compared to the untreated HepG2 cancer cells (control). #P < 0.05, compared to sorafenib treated HepG2 cancer cells.

The effect of sorafenib, bevacizumab, panitumumab, ramucirumab, sorafenib-bevacizumab, sorafenib-panitumumab and sorafenib-ramucirumab on the relative gene expression of *CASPASE3, MMP-9, VEGFR2* and *EGFR* in HepG2 cancer cells was determined by RT-PCR using *GAPDH* as a normalizer (Figs. 6, 7). The tested treatments resulted in a significant increase in the relative expression of *CASPASE3* ranging from 4.82 to 23.75 fold, with the highest increase recorded in case of bevacizumab (23.75 fold) (Fig. 6A); combinations resulted in a significant fold increase in *CASPASE3* expression compared to sorafenib single treatment except in case of sorafenib-panitumumab combination (Fig. 6A).

Tested treatments resulted in a significant decrease in the relative expression of *MMP-9* ranging from 0.9 to 0.1 fold in HepG2 cancer treated cells (Fig. 6B); the most pronounced effect was recorded in case of sorafenib-ramucirumab combination (0.1 fold). Combinations resulted in significant decrease in the relative gene expression of *MMP-9* compared to sorafenib. The effect of sorafenib, bevacizumab and their combination on the relative gene expression of *VEGFR2* in HepG2 cancer cells are shown in Fig. 7; all treatments resulted in a significant decrease in the relative expression of *VEGFR2* ranging from 0.8 to 0.4 fold (Fig. 7). Sorafenib-bevacizumab combination resulted in a significant decrease in the relative gene expression of *VEGFR2* compared to sorafenib. The effect of sorafenib, panitumumab and their combination on the relative gene expression of *EGFR* in HepG2 cancer treated cells are shown in Fig. 7. The tested treatments significantly decreased the fold expression of *EGFR* from 0.7 to 0.3 fold; combination of sorafenib with panitumumab caused a significant decrease in the relative gene expression of *EGFR* compared to either sorafenib or panitumumab.

### Effect of single and combined treatments on the protein levels of pSTAT3 and pVEGFR2 in HepG2 cancer treated cells

**Figure 8:**
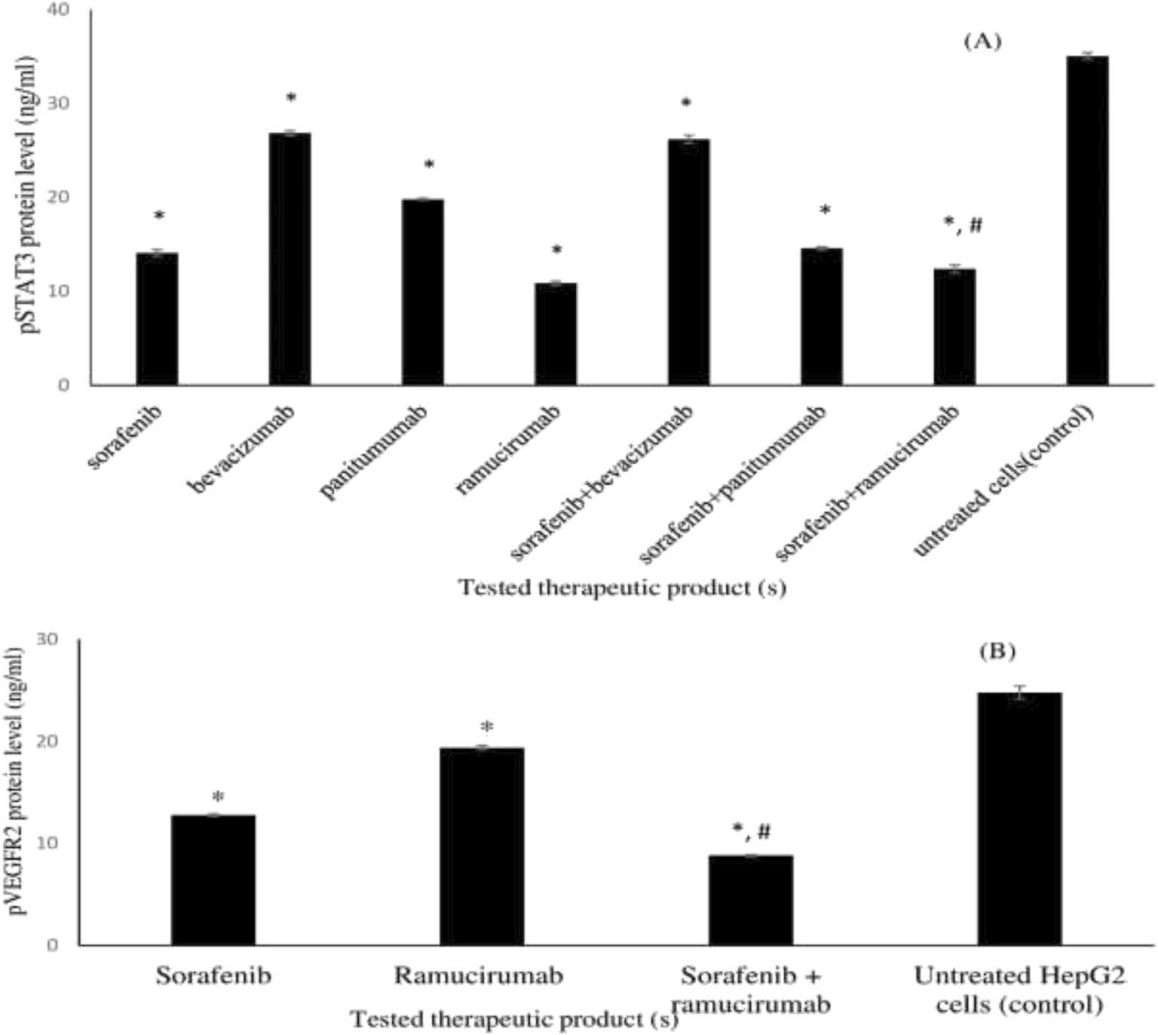
The protein levels of pSTAT3 and pVEGFR2 in HepG2 cancer treated cells. The protein levels were as follows: (**A**) protein levels of pSTAT3 upon separate and combined treatments with sorafenib, bevacizumab, panitumumab and ramucirumab; (**B**) protein level of pVEGFR2 upon separate and combined treatments with sorafenib and ramucirumab. Each value represents the mean ± SD (n = 3). *P < 0.05 compared to untreated HepG2 cancer cells (control). #P < 0.05, compared to sorafenib treated HepG2 cancer cells.

A significant decrease in pSTAT3 protein level was observed in HepG2 cancer cells treated with sorafenib, bevacizumab, panitumumab, ramucirumab and with sorafenib combined with either ramucirumab or bevacizumab, with the pronounced effect observed in case of ramucirumab treatment (0.31) (Fig. 8A and S2 Table). A significant decrease in pVEGFR2 protein level occurred in HepG2 cancer cells upon separate or combined treatments with sorafenib and ramucirumab, with the highest decrease was recorded in case of sorafenib-ramucirumab combination (0.36) (Fig. 8B and S2 Table).

### Effect of single and combined treatments on the protein expression of cleaved CASPASE3, VEGFR2 and EGFR in HepG2 cancer cells

**Figure 9:**
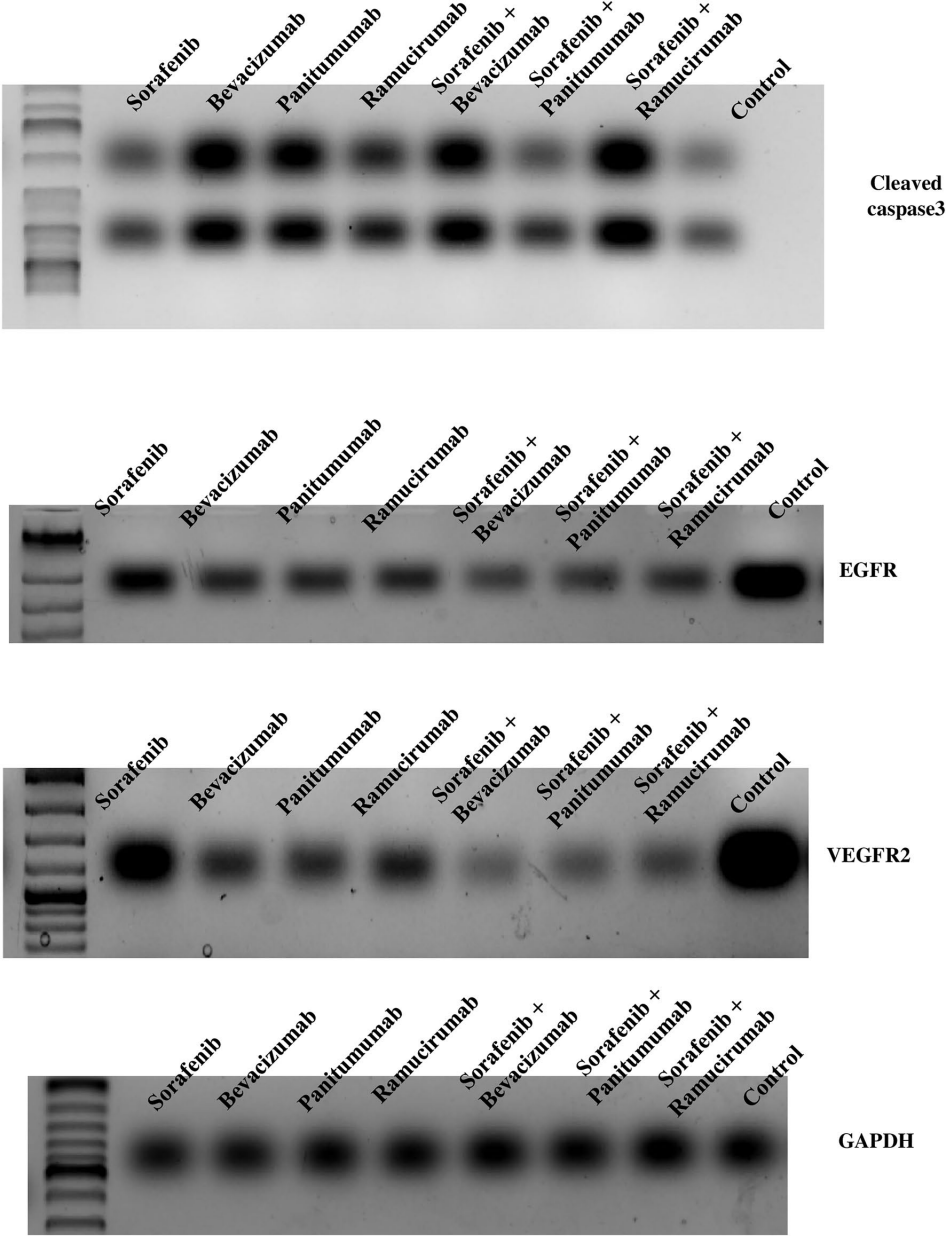
Western blot of the cleaved CASPASE-3 (≈20 kDa), VEGFR2 (≈147 kDa) and EGFR (≈180 kDa) expressed in HepG2 cancer cells following a 48 h treatment period of single and combined treatments. GAPDH (≈38 kDa) was used as a normalizer and BLUelf Prestained Protein Ladder (3.5–245 kDa) was used in all runs. Blots were cut prior to hybridization with the antibodies.

The effect of sorafenib, bevacizumab, panitumumab, ramucirumab, sorafenib- bevacizumab, sorafenib-panitumumab and sorafenib-ramucirumab on the protein expression of cleaved CASPASE3, VEGFR2 and EGFR in HepG2 cancer cells was determined by western blot using GAPDH as a normalizer (Fig. 9). All tested treatments resulted in an increase in the abundance of cleaved CASPASE3 ranging from 2.3 to 8.9 fold, with the highest increase recorded in case of bevacizumab (8.9 fold) followed by sorafenib-ramucirumab (8.5 fold). A decrease in the expression of VEGFR2 (0.69–0.13 fold) and EGFR (0.56–0.22 fold) was observed with all tested treatments. Combination treatments showed more pronounced fold decrease in VEGFR2 and EGFR compared to sorafenib single treatment.

## Discussion

Hepatocellular carcinoma is one of the world’s top ten solid malignant tumors in terms of morbidity and mortality^37^ and ranks the fourth in Egypt^38^. Worldwide, Egypt is among the top 25 countries with the highest rates of liver cancer^39^. The key signal transduction pathways in the pathogenesis of hepatocellular carcinoma include: VEGF, EGFR, Ras/Raf/Mek/Erk, phosphoinositide 3-kinase/Akt, mTOR, HGF/c-Met, Wnt, and Hedgehog signaling cascades^40^. Consequently, drugs that selectively target these pathways might, have anticancer therapeutic potential. Sorafenib is a molecular targeted agent approved, as the first-line systemic treatment for advanced HCC, to improve the overall survival in patients^41^. It induces apoptosis in cancer cells, inhibits tumor angiogenesis and cell proliferation^10^. Other tyrosine kinase inhibitors, including erlotinib, brivanib, sunitinib, and linifanib were tested as first-line treatments for HCC, but they didn’t show any advantage over sorafenib^10^. Sorafenib has severe adverse effects that compromises the continuity of treatment^42^. The simultaneous inhibition of multiple signaling pathways is a key approach for the management HCC^43^. Although sorafenib has antiangiogenic effects, the complexity of angiogenesis suggests that it cannot completely block the formation of tumor microvessels^44^. The redundancy of angiogenic mechanisms may contribute to drug resistance through the activation of alternative pro-angiogenic pathways. Thus, combining sorafenib with other antiangiogenic agents, with different targets, may improve the efficacy of sorafenib monotherapy and minimize the arising of drug resistance^44^. Thus, drug combinations offer the opportunity to reduce sorafenib doses without compromising its effect^43^. In this study, we tested the effect of sorafenib combination with an anti-VEGF (bevacizumab), anti-VEGFR2 (ramucirumab) and anti-EGFR (panitumumab) on HepG2 cancer cells viability, regulation of cell cycle, apoptosis, expression of VEGFR2, EGFR, *MMP-9* and CASPASE3 and the levels of pVEGFR2 and pSTAT3 proteins compared to sorafenib.

In agreement with previous studies, sorafenib treatments for 24, 48 and 72 h exhibited cytotoxic potential towards HepG2 cancer cells^45–48^. Both panitumumab and ramucirumab caused a gradual decrease in HepG2 cancer cells’ viability which was more pronounced at ≥ 500 µg/mL concentrations in the 48 h treatment period and at ≥ 31.25 µg/mL concentrations in the 72 h treatment period. Similar responses were previously reported with panitumumab on colorectal cancer cell line after 72 h^49^; ramucirumab on HCC carcinoma and gastric cell line after 48 h^26,27^. A slight decrease in cell proliferation was observed with bevacizumab at prolonged treatment period (72 h) with high tested concentrations (≥ 1 mg/mL). Similar responses were reported for bevacizumab on head and neck squamous cell carcinoma^50^, on glioma cells after 24 and 72 h^51^, and on human retinoblastoma after 48 h treatment period^52^. Addition of panitumumab or ramucirumab to sorafenib, was advantageous in terms of reducing the IC50 of sorafenib. Similar approaches testing combinations of sorafenib with anti-VEGFR2 and anti-EGFRvIII mAbs showed *in-vitro* inhibitory effects on PLC/PRF/5 and HepG2 cancer cell lines^26,53^. On the other hand, adding bevacizumab to sorafenib had nearly no effect on sorafenib’s IC_50_ following a 48 h treatment period with an unexpected increase in sorafenib IC_50_ following the 24 h treatment period. Treatment of HepG2/C3A cancer cell line with bevacizumab 5 ng/mL and 100 μg/mL for 48 h was previously reported to increase telomerase activity which in turn resulted in the overexpression of VEGFR1 and VEGFR2^54^. Our findings regarding the antagonistic effect of sorafenib-bevacizumab combination on cell viability could be due to the neutralization of the effect of VEGFR inhibition by sorafenib with bevacizumab overexpression of VEGFR. This could also explain the ineffectiveness observed in phase l/ll randomized trials of sorafenib-bevacizumab combinations in treating HCC^55^.

We examined cell cycle progression in HepG2 cancer treated cells by DNA flow cytometry. Cell cycle arrest in G2/M was recorded in case of single treatments and sorafenib-panitumumab combination. This finding was in line with the reported ability of sorafenib to induce cell cycle arrest in cancer cells^56,57^, but on the contrary bevacizumab and ramucirumab are reported to induce cell cycle arrest in G1/S^17,58^. Bevacizumab causes a significant accumulation of the percentage of cells in the G0/G1 phase, while it decreases the fraction of cells in the G2 and M phases in HCC and choroidal endothelial cells in a concentration dependent effect^17^. However, in another study, ramucirumab was ineffective in inhibiting the progression from the G2/M phase to the subsequent G0/G1phase of cell cycle of gastric cancer^27.^

Significant cell accumulation in the preG1 and G0/G1 phases following treatment with sorafenib combination with bevacizumab (52. 6%) and ramucirumab (57.2%) indicates improved cell cycle arrest and enhanced cell death. These findings support the enhanced inhibitory effect of sorafenib-ramucirumab combination observed in HepG2 cancer treated cell line. Cell growth is a result of the balance between proliferation and apoptosis^3^. Mechanistically, tumor inhibition by combination therapies may result from an increased capacity to induce apoptosis^57,59^. We recorded an increase in apoptosis, especially late apoptosis, with combined treatments of sorafenib with either bevacizumab (1.9 fold) or ramucirumab (1.8 fold), compared to single sorafenib treatment. This agrees with previous studies that reported a pronounced effect on apoptosis following the addition of ramucirumab to either regorafenib or sorafenib^26^. However, sorafenib-panitumumab didn’t enhance apoptosis which disagrees with a previous study that reported a significant increase in the number of apoptotic cells following treatment with a combination of sorafenib and an anti-EGFRvIII^53^. These findings were confirmed by assessing the relative expression of *CASPASE3* and the abundance of the pro-apoptotic marker, cleaved CASPASE-3, in HepG2 cancer treated cells. CASPASES are the main executors of the apoptotic process, as they carry out the execution of cellular demolition, thus permitting their involvement in cancer treatment^60,61^. We reported an overexpression in *CASPASE3* and cleaved CASPASE-3 in HepG2 cancer cells treated with sorafenib combinations with either bevacizumab or ramucirumab compared to sorafenib. This is in accordance with previous studies reporting the significant overexpression of *CASPASE3* following the use of sorafenib-ramucirumab combinations^17,61^. In our study, this inhibitory activity on HepG2 cancer cells was supported by an inhibition in the expression *MMP-9*, VEGFR2 and EGFR. A down-regulated expression of VEGFR2 and EGFR was reported in HepG2 cancer cells treated by single and combined treatments of sorafenib and ramucirumab^26,62^. Degradation of the extracellular matrix by MMP releases proangiogenic compounds, such as vascular endothelial growth factor (VEGF) and integrins^63^. MMP9 has been identified as a biomarker in various cancers, mainly when tumor expression is considered^63^. All single treatments down-regulated the expression of *MMP-9*, VEGFR2 and EGFR. This is in agreement with previous studies that reported down regulation in the expression of *MMP-9* by sorafenib^64^, and panitumumab^65^; VEGFR by ramucirumab^26,66^, sorafenib^64^, and bevacizumab^67^ and EGFR by panitumumab^13^ and sorafenib^48^. Combining sorafenib with panitumumab significantly down regulated the expression of each of VEGFR and EGFR in HepG2 cancer treated cells. This is in agreement with previous studies that highlighted the synergistic antitumor activity of sorafenib combinations with EGFR inhibitors in various tumors including human non–small cell lung cancer, colorectal cancer, and HCC^53,68^. Both VEGF and EGF share common downstream signaling pathways and may function exclusively of one another during oncogenes so, dual inhibition of EGFR and VEGFR might yield greater antitumor activity^69^.

Signal transducer and activator of transcription-3 (STAT3) is in the research spot asan oncogenic signaling molecule. It plays a critical role in transcriptional regulation of genes that are involved in tumor cell proliferation, survival, migration and invasion into the extracellular matrix^53,70^; constitutive activation of STAT3 is observed in 72.4% of human HCC^58^. It is also involved in the invasion, metastasis during tumor progression and angiogenesis; has anti-apoptosis and inflammatory response^1,17,48^ . Single treatments with sorafenib, bevacizumab, panitumumab and ramucirumab significantly decreased pSTAT3 protein level in HepG2 cancer cells. Similar inhibition of STAT3 signaling in some cancers, including HCC was reported when treating with sorafenib^53,62^, bevacizumab^71,72^, ramucirumab^26,67^, panitumumab^71,73,74^, and a combination of sorafenib with an anti-EGFRvIII^53^. Sorafenib-ramucirumab combination significantly reduced pSTAT3 protein level compared to sorafenib which is beneficial in treating HCC. A recent phase 3 clinical trial showed an improved overall survival for treatment with ramucirumab compared to placebo in patients with hepatocellular carcinoma and α-fetoprotein concentrations of at least 400 ng/mL who had received ramucirumab treatment after prior treatment with sorafenib^42,75^. An expansion cohort of REACH-2 represents a non-sorafenib sequencing study in patients with advanced HCC. The safety/efficacy profile of ramucirumab following a non-sorafenib based systemic therapy was consistent with that observed in patients who received prior sorafenib treatment^76^. Regarding safety, promising combination therapies containing ramucirumab are likely to pave the way for the future effective treatment of HCC^77^.

## Conclusions

The present study demonstrates the synergistic interaction of ramucirumab with sorafenib to invoke a strong anticancer activity against hepatocellular carcinoma by inducing apoptosis; inhibiting cell growth/proliferation; upregulating the expression of *CASPASE3*; downregulating the expression of *MMP-9*, EGFR and VEGFR2 and decreasing pSTAT3 and pVEGFR2 protein levels in HepG2 cancer treated cells. Downregulation of MMP9, EGFR, VEGFR and STAT3 in HepG2 cancer cells could appear to play a role in tumor invasion and angiogenesis and to mediate the tumor microenvironment. This is in addition to decreasing the IC50 of sorafenib that could allow lowering sorafenib doses and a possible decrease in its toxic side effects. This suggests that combining ramucirumab with the sorafenib could be an attractive strategy for treating patients with advanced hepatocellular carcinoma. Further confirmation of the enhanced activity of ramucirumab-sorafenib combination should be tested in Huh-7 hepatocellular carcinoma cell lines and normal cell lines.

## Supplementary Information

Supplementary Information.

## Data Availability

All data generated or analyzed during this study are included in the manuscript and Supporting information.
