# Supplementary material for: Ramucirumab combination with sorafenib enhances the inhibitory effect of sorafenib on HepG2 cancer cells
Source: Sci Rep. 2022 Oct 25;12:17889. doi: 10.1038/s41598-022-21582-w (PMC9596484; doi:10.1038/s41598-022-21582-w)
Supplement: Supplementary file 1 — Supplementary Information. [file 41598_2022_21582_MOESM1_ESM.docx]

**Ramucirumab combination with sorafenib enhances the inhibitory effect of sorafenib on HepG2 cancer cells**

Amna Mohamed Taha^1^, Mohammad Mabrouk Aboulwafa^2,3*^, Hamdallah Hafez Zedan^4^, Omneya Mohamed Helmy^4^

1 Egyptian Drug Authority, Wezaret Alzera'h St., Doki, Cairo, Egypt

2 Department of Microbiology and Immunology, Faculty of Pharmacy, Ain Shams University, Al Khalifa Al Ma'moun St., Abbassia, Cairo, Egypt

3 Faculty of Pharmacy, King Salman International University, Ras-Sedr, South Sinai, Egypt

4 Department of Microbiology and Immunology, Faculty of Pharmacy, Cairo University, Kasr El-Aini St., Cairo 11562, Egypt

*** Corresponding author:** Prof. Dr. Mohammad M. Aboulwafa

Email: maboulwafa@yahoo.com; [maboulwafa@pharma.asu.edu.eg](mailto:maboulwafa@pharma.asu.edu.eg); mohammad.aboulwafa@ksiu.edu.eg

Telephone No.: +2 01002350371

Address: Department of Microbiology & Immunology, Faculty of Pharmacy, Ain Shams University, Al Khalifa Al Ma'moun St., Abbassia, Cairo, Egypt; Faculty of Pharmacy, King Salman International University, Ras-Sedr, South Sinai, Egypt

**S1 table. Combination Index values for each combined treatment calculated by CompuSyn software**

| Dose of Sorafenib | Dose of each mabs (µg/ml) | CI of ramucirumab | CI of bevacizumab | CI of panitumumab |
| --- | --- | --- | --- | --- |
| (µM) |  |  |  |  |
| 25 | 62.5 | 0.82 | 0.99 | 0.82 |
| 12.5 |  | 0.32 | 0.60 | 0.39 |
| 6.25 |  | 1.79 | 1.39 | 1.08 |
| 3.125 |  | 6.66 | 2.50 | 1.97 |
| 1.56 |  | 1.64 | 1.21 | 1.31 |
|  |  |  |  |  |
| 25 | 125 | 0.67 | 0.99 | 0.83 |
| 12.5 |  | 0.33 | 0.58 | 0.35 |
| 6.25 |  | 1.30 | 1.50 | 1.05 |
| 3.125 |  | 3.57 | 2.43 | 1.47 |
| 1.56 |  | 1.75 | 1.13 | 0.71 |
|  |  |  |  |  |
| 25 | 250 | 0.83 | 0.99 | 0.80 |
| 12.5 |  | 0.46 | 0.60 | 0.50 |
| 6.25 |  | 0.25 | 1.18 | 0.28 |
| 3.125 |  | 0.14 | 0.71 | 0.25 |
| 1.56 |  | 0.97 | 1.11 | 0.72 |
|  |  |  |  |  |
| 25 | 500 | 0.61 | 0.99 | 0.83 |
| 12.5 |  | 0.35 | 0.59 | 0.50 |
| 6.25 |  | 0.19 | 1.20 | 0.25 |
| 3.125 |  | 0.13 | 0.72 | 0.24 |
| 1.56 |  | 1.01 | 1.08 | 0.64 |

**S2 table Raw data of protein levels of pSTAT3 in HepG2 cancer treated cells**

| Therapeutic progucts | Measurement | Conc | Average |
| --- | --- | --- | --- |
| Sorafenib | 0.472 | 14.74163 | 14.10367 |
|  | 0.455 | 13.92823 |  |
|  | 0.449 | 13.64115 |  |
| Bevacizumab | 0.732 | 27.18182 | 26.84689 |
|  | 0.718 | 26.51196 |  |
|  | 0.725 | 26.84689 |  |
| Panitumumab | 0.579 | 19.86124 | 19.82935 |
|  | 0.581 | 19.95694 |  |
|  | 0.575 | 19.66986 |  |
| Ramucirumab | 0.392 | 10.91388 | 10.86603 |
|  | 0.383 | 10.48325 |  |
|  | 0.398 | 11.20096 |  |
| Sorafenib+Bevacizumab | 0.453 | 13.83254 | 14.58214 |
|  | 0.481 | 15.17225 |  |
|  | 0.472 | 14.74163 |  |
| Sorafenib+Panitumumab | 0.417 | 12.11005 | 12.38118 |
|  | 0.428 | 12.63636 |  |
|  | 0.423 | 12.39713 |  |
| Sorafenib+Ramucirumab | 0.723 | 26.7512 | 26.20893 |
|  | 0.713 | 26.27273 |  |
|  | 0.699 | 25.60287 |  |
| Untreated cells (control) | 0.346 | 35.2 | 35.06667 |
|  | 0.372 | 35.1 |  |
|  | 0.366 | 34.9 |  |

**S3 table Raw data of protein levels of pVEGFR2 in HepG2 cancer treated cells**

| **Therapeutic products** | **Measurement** | **Conc** | **Average** |
| --- | --- | --- | --- |
| **Sorafenib** | 0.459 | 12.66 | 12.77 |
|  | 0.462 | 12.76 |  |
|  | 0.466 | 12.88 |  |
| **Ramucirumab** | 0.674 | 19.63 | 19.4 |
|  | 0.666 | 19.36 |  |
|  | 0.662 | 19.22 |  |
| **Sorafenib+Ramucirumab** | 0.329 | 8.79 | 8.828 |
|  | 0.334 | 8.933 |  |
|  | 0.328 | 8.761 |  |
| **Untreated cells (control)** | 0.806 | 24.18 | 24.81 |
|  | 0.841 | 25.42 |  |
|  | 0.824 | 24.82 |  |


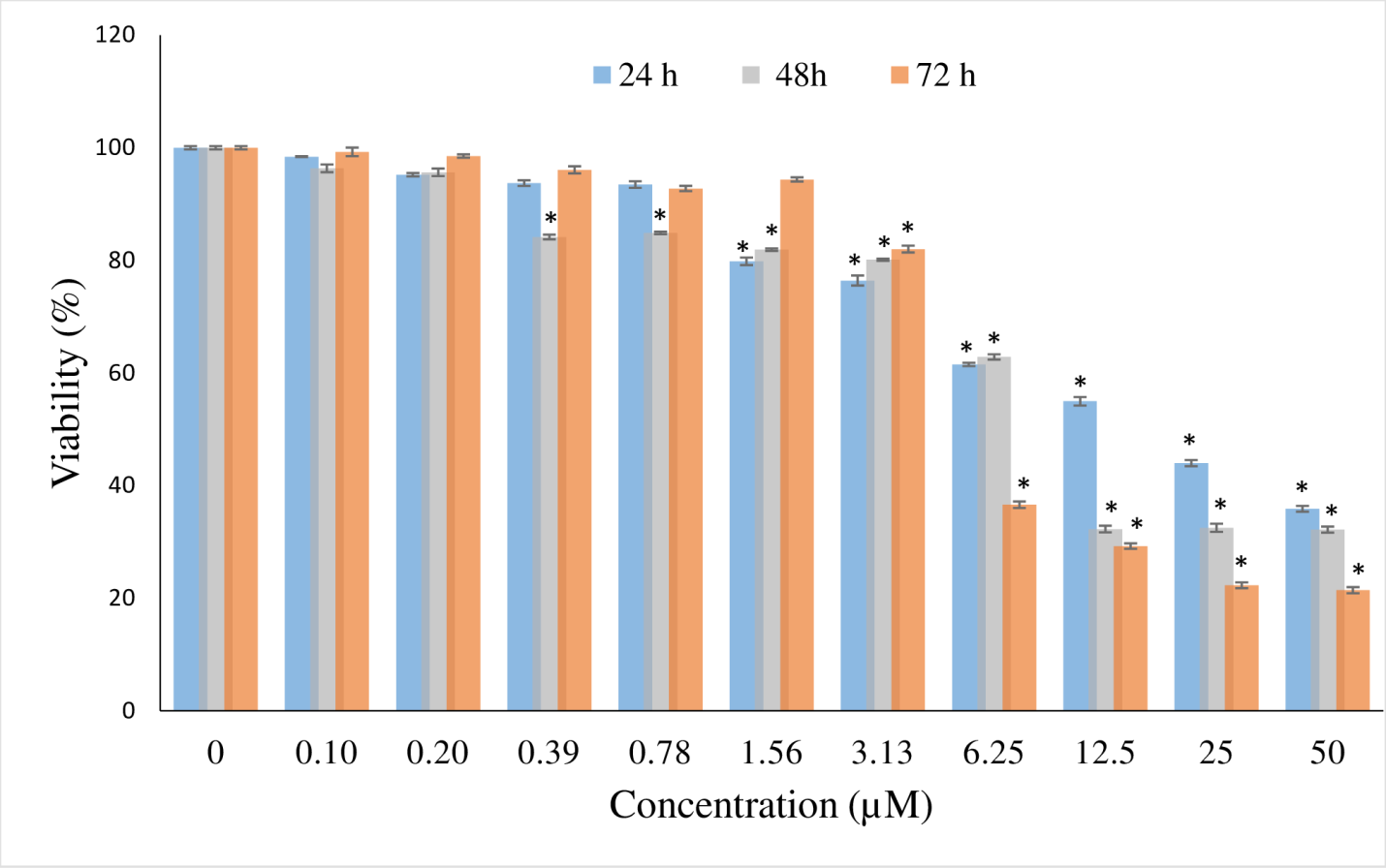


**S1 Fig. Effect of different concentrations of sorafenib on the viability of HepG2 cancer cells following 24, 48 and72 hours treatment periods measured by MTT Assay.** Each value represents the mean ± SD (n = 3). *, P < 0.05


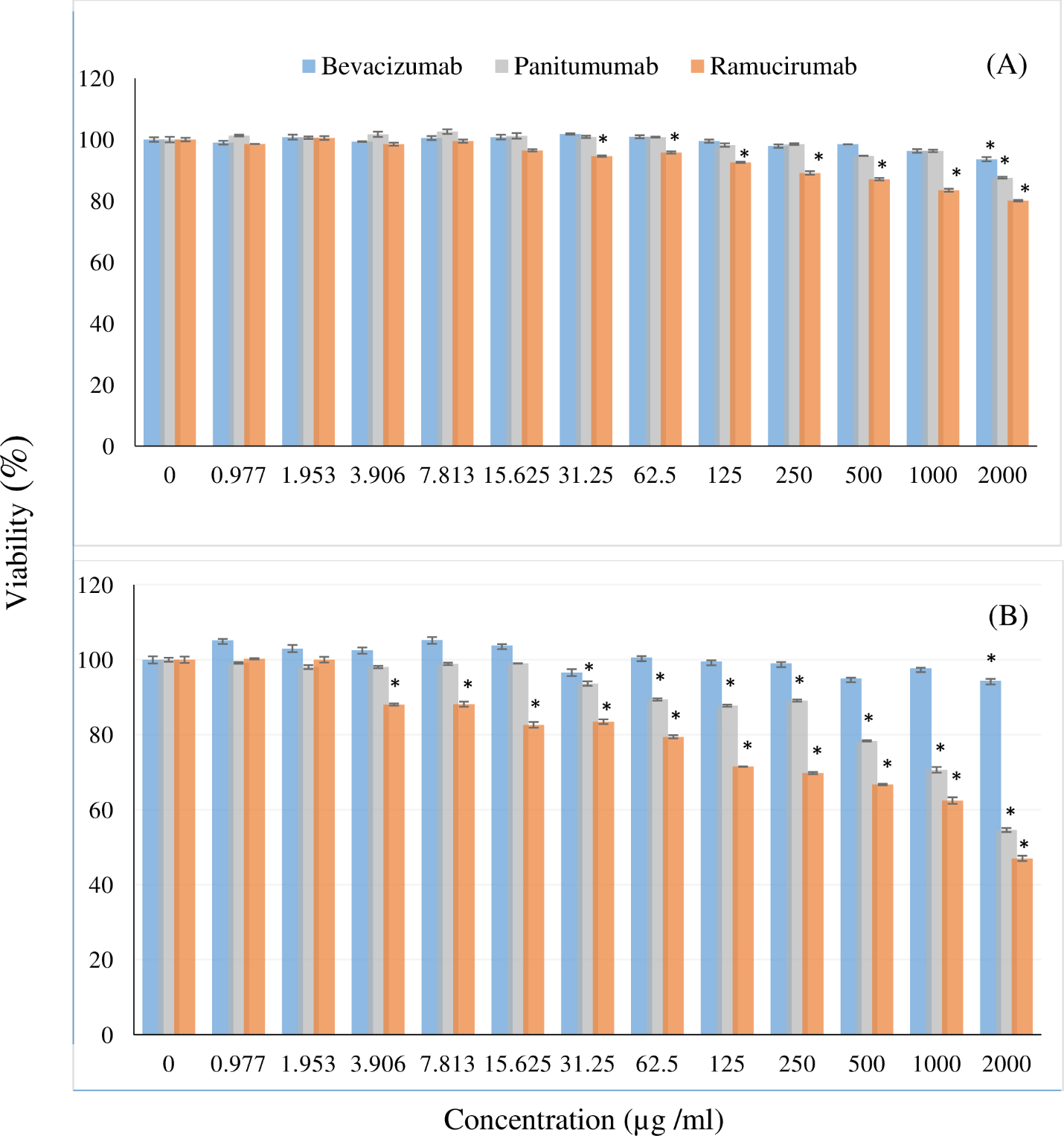


**S2 Fig. Effect of different concentrations of bevacizumab, panitumumab and ramucirumab on the viability of HepG2 cancer cells measured by MTT assay. (A) following 48 hours treatment; (B) following 72 hours treatment.** Each value represents the mean ± SD (n = 3). *, P < 0.05


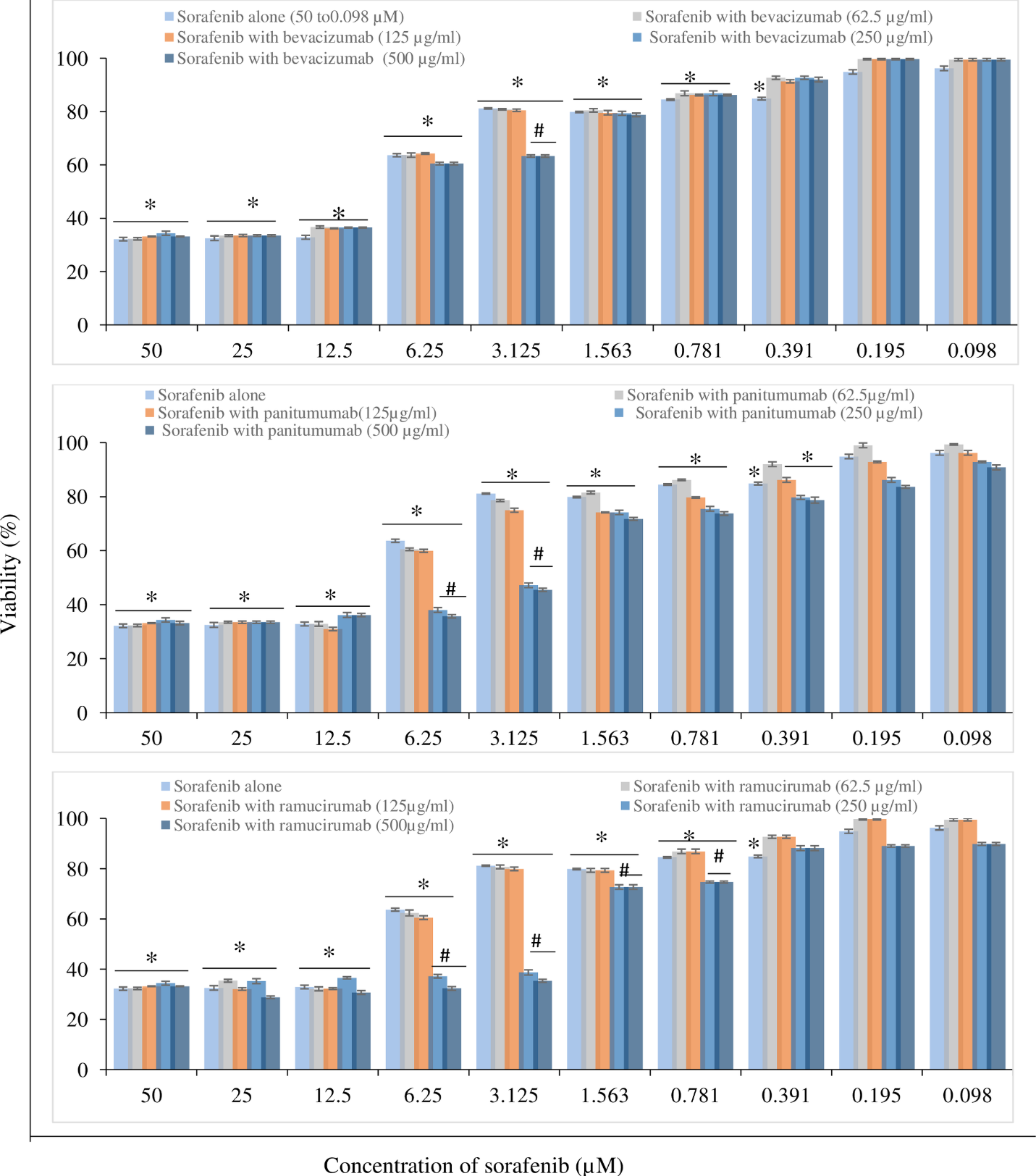


**S3 Fig. Effect of different concentrations of sorafenib in presence and absence of bevacizumab, panitumumab and ramucirumab on the viability of HepG2 cancer cells following 48 hours periods measured by MTT assay.** Each value represents the mean ± SD (n = 3). *, P < 0.05


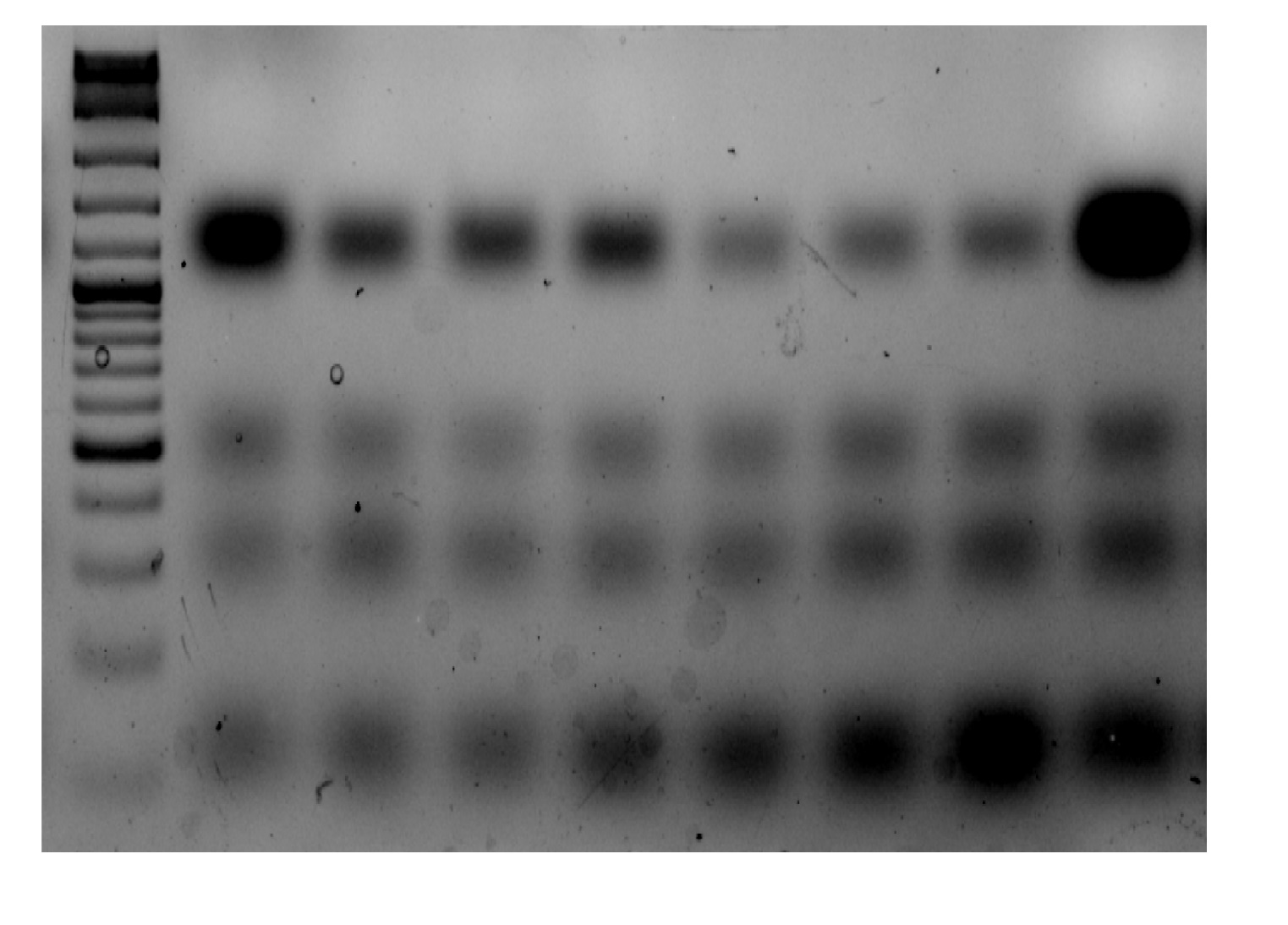


**VEGFR2**

**Sorafenib**

**Control**

**Sorafenib+ Ramucirumab**

**Sorafenib + Panitumumab**

**Sorafenib + bevacizumab**

**panitumumab**

**Ramucirumab**

**Bevacizumab**

S4 Fig. Full-length original blots of Fig 9





S5 Fig. Full-length original blots of Fig 9


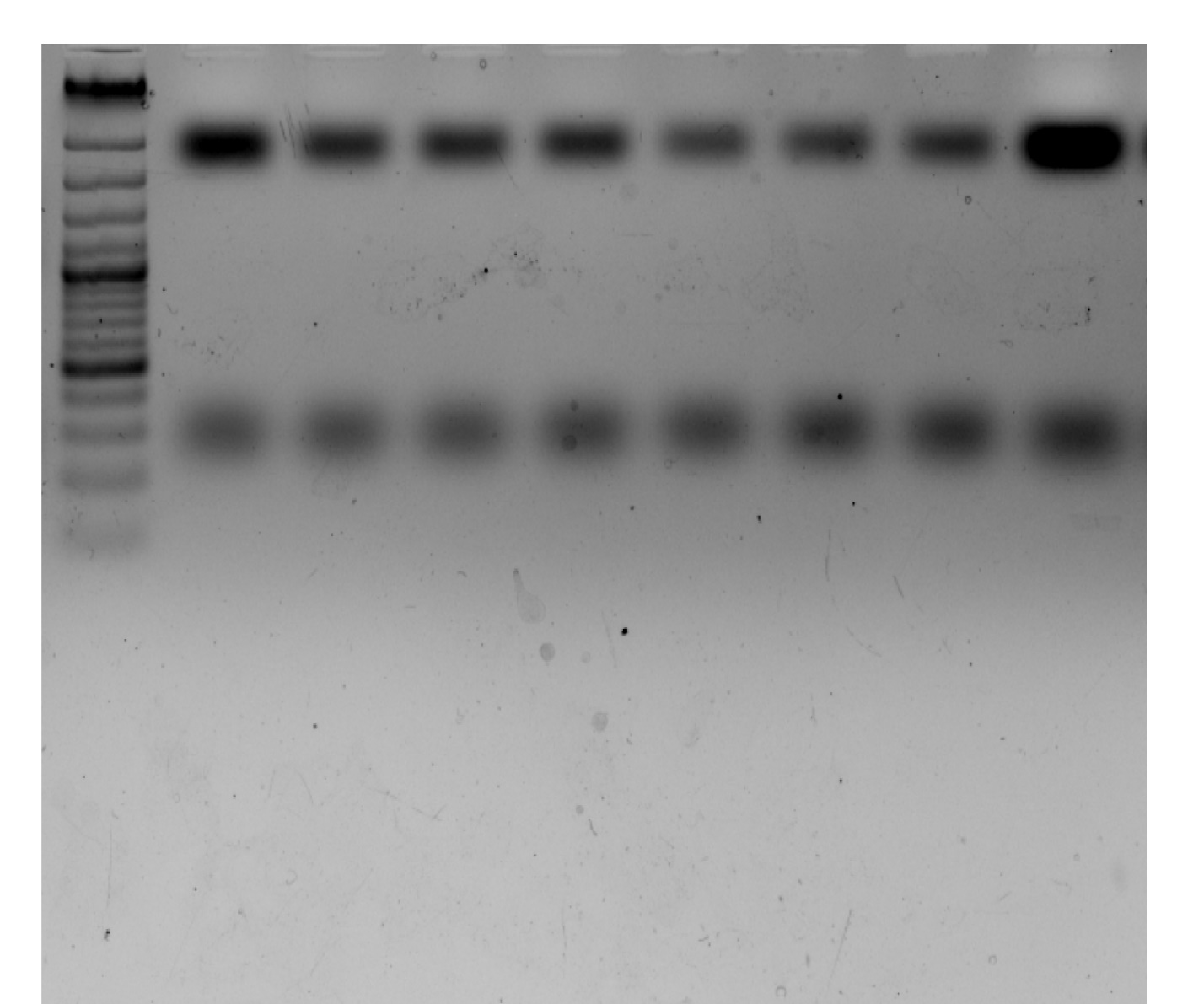


**Control**

**Sorafenib + Ramucirumab**

**Sorafenib + Panitumumab**

**Sorafenib + Bevacizumab**

**Panitumumab**

**Ramucirumab**

**Bevacizumab**

**Sorafenib**

**EGFR**

S6 Fig. Full-length original blots of Fig 9


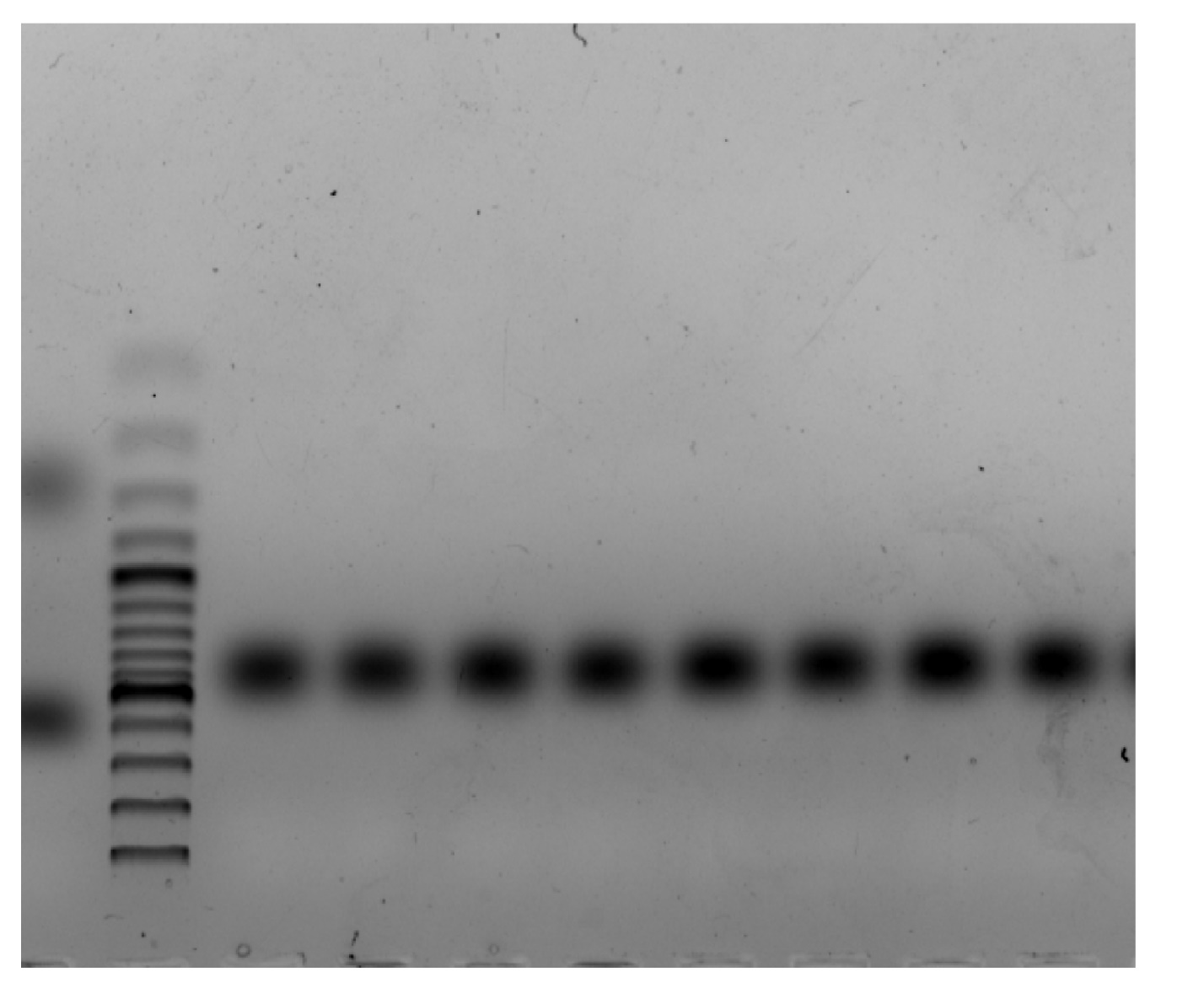


**GAPDH**

S7 Fig. Full-length original blots of Fig 9
